# Complete uterine septum, double cervix, and longitudinal vaginal septum: an integrated approach for one-stop diagnosis and ultrasound-guided endoscopic treatment

**DOI:** 10.52054/FVVO.15.2.077

**Published:** 2023-06-30

**Authors:** F Pozzati, M Mirandola, G Topozouva, L Parodi, A Carla Testa, G Scambia, U Catena

**Affiliations:** Dipartimento di Scienze della Salute della Donna, del Bambino e di Sanità Pubblica, Fondazione Policlinico Universitario A. Gemelli IRCCS, U.O.C. di Ginecologia Oncologica, Rome, Italy; Università Cattolica del Sacro Cuore, Rome, Italy

**Keywords:** Congenital anomaly, uterine malformation, ultrasound, Hysteroscopy, U2bC2V1

## Abstract

**Background:**

A complete uterine septum, double cervix and vaginal septum is a complex and rare congenital genital tract anomaly. The diagnosis is often challenging and based on the combination of different diagnostic techniques and multiple treatment steps.

**Objective:**

To propose a combined one-stop diagnosis and an ultrasound-guided endoscopic treatment of complete uterine septum, double cervix, and longitudinal vaginal septum anomaly.

**Materials and Methods:**

Stepwise demonstration with narrated video footage of an integrated approach management of a complete uterine septum, double cervix and vaginal longitudinal septum treated by expert operators combining minimally invasive hysteroscopy and ultrasound. The patient was 30 years old and was referred to our clinic because of dyspareunia, infertility and the suspicion of a genital malformation.

**Results:**

A one-stop complete evaluation of uterine cavity, external profile, cervix, and vagina was made through 2D, and 3D ultrasound combined with hysteroscopic assessment and a U2bC2V1 malformation (according to ESHRE/ESGE classification) was diagnosed. The procedure consisted in a totally endoscopic removal of the vaginal longitudinal septum and the complete uterine septum, starting the uterine septum incision from the isthmic level, and sparing the two cervices, under transabdominal ultrasound guidance. The ambulatory procedure was performed in the Digital Hysteroscopic Clinic (DHC) CLASS Hysteroscopy in Fondazione Policlinico Gemelli IRCCS of Rome - Italy, under general anaesthesia (laryngeal mask).

**Main outcomes:**

Surgical time of procedure was 37 minutes; no complications occurred; patient was discharged three hours after the procedure; the hysteroscopic office control after 40 days showed a normal vagina and a normal uterine cavity with two normal cervices.

**Conclusion:**

An integrated ultrasound and hysteroscopic approach allows an accurate one-stop diagnosis and a totally endoscopic treatment option for complex congenital malformations using an ambulatory model of care with optimal surgical results.

## Learning objective

To propose a combined minimally invasive one- stop diagnosis (Campo et al., 2018) and treatment of complete uterine septum, double cervix, and vaginal septum anomaly, classified as U2bC2V1 according to ESHRE/ESGE classification (Grimbizis et al., 2013), under transabdominal ultrasound guidance.

## Introduction

The incidence of congenital uterine anomalies in the general population is estimated to be 0.001%- 10% (Louden et al., 2015). The combination of uterine septum, double cervix and vaginal septum is a complex anomaly of the genital tract whose incidence cannot be estimated because of the lack of cases described in literature. Affected women often complain of dyspareunia and might experience infertility, recurrent miscarriages, and obstetric complications. Diagnosis of this rare malformation can be challenging and often requires multiple diagnostic steps and techniques. In the past, the diagnosis was obtained combining laparoscopy and hysteroscopy. Currently, magnetic resonance and ultrasound are often used to obtain a diagnosis, even if this complex malformation is frequently misdiagnosed (Ignatov et al., 2008; Sejelfot et al., 2020; Seet et al., 2015; Wang et al., 2009; Lin et al., 2009; Tomaževič et al., 2010).

Surgical treatment is not always indicated; there is still lack of evidence on the advantages of septum removal on perinatal outcomes, even though, in selected patients, surgical treatment leads to increased pregnancy rate (Noventa et al., 2022). In case of U2bC2V1 anomaly, surgical treatment, if indicated, is not standardised (Parodi et al., 2022). Only case reports and retrospective case series are reported in the literature. The treatment of the uterine septum can be performed with different instruments and the access to the septate uterine cavity can be eased using different techniques (Di Spiezio Sardo et al., 2021; Parodi et al., 2022). After incision of the uterine septum, the removal of the septum itself was proposed by Fascilla et al. (2020). This technique allowed better post-operative outcomes, and the histological evidence of atypical muscular layer in the septum (Fascilla et al., 2020).

The double cervix differs from the unique cervix with a cervical septum (Ludwin et al., 2013); the cervical septum can be surgically treated with satisfying outcomes (Parsanezhad et al., 2006), whereas the double cervix has no surgical indications (Ludwin et al., 2013).

A longitudinal vaginal septum can be resected in symptomatic patients, presenting with dyspareunia (Grynberg et al., 2012), with many surgical techniques described.

Due to the lack of precise diagnostic criteria and standardised surgical techniques to treat U2bC2V1 patients, potential obstetric and perinatal outcomes are not always clear (Rikken et al., 2021; Di Spiezio Sardo et al., 2021; Parodi et al., 2022).

## Patients and method

This is a stepwise demonstration with narrated video footage of a one-stop diagnosis (Campo et al., 2018) and a totally endoscopic ultrasound- guided treatment of a fertile 30-year-old patient affected by a complete uterine septum, double cervix, and vaginal longitudinal septum. She was referred to our clinic because of dyspareunia, infertility, and a suspicion of complex malformation. The diagnosis was made with an integration of transvaginal ultrasound and diagnostic hysteroscopy made by an expert operator in a one-stop office procedure (Campo et al., 2018). With 2D ultrasound, the presence of a uterine septum was suspected in transverse scan. After 3D reconstruction we confirmed the convex external profile of the uterus and the presence of a complete uterine septum reaching the internal uterine orifice and dividing the uterine cavity in two hemi-cavities. Measurement of the septum length was possible on 3D reconstruction, as well as the confirmation of the presence of two cervices. The office hysteroscopy showed the presence of a complete longitudinal non- obstructing vaginal septum and confirmed the presence of two cervices and two uterine hemi- cavities. No communication between the two hemi-cavities was observed.

The surgical treatment was performed according to an ambulatory model of care (Carugno et al., 2021) in the Digital Hysteroscopic Clinic (DHC) CLASS Hysteroscopy in Fondazione Policlinico Gemelli IRCCS of Rome - Italy (Campo et al., 2018), under general anaesthesia (laryngeal mask). A minimally invasive endoscopic technique using a 15fr bipolar mini-resectoscope under ultrasound transabdominal guidance was performed as follows: 1) complete vaginoscopic precise incision of the longitudinal vaginal septum with a Collins loop; 2) incision of the uterine septum with a Collins loop at the level of its minor width in proximity of the internal uterine orifice, under transabdominal ultrasound guidance, until the achievement of the communication of the two hemi-cavities. Thanks to the support of transabdominal ultrasound guidance, the mini- resectoscope is identified inside the uterus and the region of minor width of the uterine septum to be incised was easily recognised. After the initial cutting of the septum in one hemi-cavity, a hyperechoic dot indicates the correct point of incision in the other hemi-cavity; 3) complete incision of the uterine septum with the Collins loop to reach the interostial plane leaving at the end of the procedure a total residual fundal thickness of 12 mm, measured ultrasonographically (Bajka and Badir, 2017; Di Spiezio Sardo et al., 2016); 4) removal of the redundant tissue at the level of the anterior and posterior uterine wall with a 15 Fr 90-degrees angled loop (Fascilla et al., 2020). Double cervix was left unmodified (Ludwin et al., 2013).

## Results

The patient was discharged 3 hours after the procedure. No complications occurred. Surgical time of procedure was 37 minutes. Hysteroscopic office control 40 days after the procedure showed a normal vagina with no residual septum, two cervices and a unique normal uterine cavity. Cold fundal cuts with 5 Fr scissors were performed to refine the uterine fundus (Catena, 2022). No intrauterine adhesions were described. After the procedure the total myometrial fundal thickness was of 12 mm at ultrasound 3D reconstruction.

## Discussion

In this video article we proposed a step-by-step procedure of removal of uterine septum up to the isthmic level sparing of the two cervices and resection of longitudinal vaginal septum in a patient diagnosed with U2bC2V1 anomaly (Grimbizis et al., 2013).

The surgery was concluded in a one-stop endoscopic procedure under transabdominal ultrasound guidance, according to an ambulatory model of care (Carugno et al., 2021). Transabdominal ultrasound guidance eased the access of the hysteroscope and permitted a complete vision of both the uterine hemi-cavities and the external uterine margin. The best site of septum incision was precisely indicated to the surgeon and false paths were avoided. Nevertheless, ultrasound guidance performance can be limited by intestinal meteorism, elevated BMI and a retroverted uterus.

The strengths of our technique are the possibility to diagnose and treat complex malformations in the same calendar day (the patient is discharged 3 hours after the procedure), avoiding multiple steps; the achievement of optimal surgical results with a minimally invasive approach avoiding major complications and the possibility for the patient to try to achieve pregnancy 40 days after the hysteroscopic control.

Major limitation of our paper is that it is a single case representation, and more cases should be gathered to describe appropriate results. There is still a lack of evidence that surgical treatment of U2bC2V1 malformations leads to better obstetric and perinatal outcomes, even though in symptomatic patients, an increased pregnancy rate is described (Noventa et al., 2022); surgical management should be proposed only in selected patients.

## Conclusions

Correct diagnosis of complex congenital Mullerian anomalies is crucial for identification of patients needing surgical treatment. One-stop diagnosis using an ambulatory model of care can be safely performed in a digital hysteroscopic clinic with optimal surgical results. Further studies are needed to standardise the technique and to evaluate the potential fertility and obstetric outcomes of these patients.

## Video scan (read QR)

https://vimeo.com/esge/pozzati-et-al?share=copy

**Figure:**
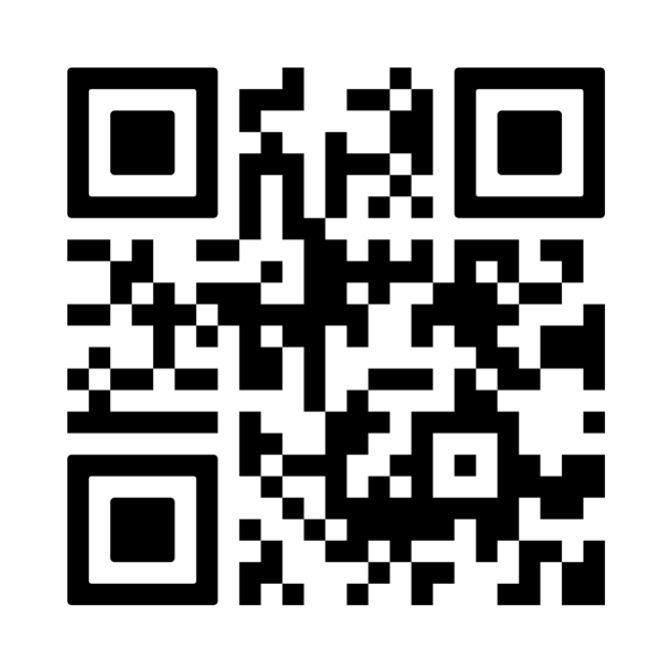


## References

[1] Bajka M, Badir S (2017). Fundus thickness assessment by 3D transvaginal ultrasound allows metrics-based diagnosis and treatment of congenital uterine anomalies.. Ultraschall Med.

[2] Campo R, Santangelo F, Gordts S (2018). Outpatient hysteroscopy.. Facts Views Vis Obgyn.

[3] Carugno J, Grimbizis G, Franchini M (2021). International Consensus Statement for recommended terminology describing hysteroscopic procedures.. Facts Views Vis Obgyn.

[4] Catena U (2022). Surgical treatment of the septate uterus.. Hysteroscopy Newsletter.

[5] Di Spiezio Sardo A, Manzi A, Zizolfi B (2021). The step-by-step hysteroscopic treatment of patients with vaginal and complete uterine septum with double cervix (U2bC2V1).. Fertil Steril.

[6] Di Spiezio Sardo A, Zizolfi B, Bettocchi S (2016). Accuracy of hysteroscopic metroplasty with the combination of presurgical 3-dimensional ultrasonography and a novel graduated intrauterine palpator: a randomized controlled trial.. J Minim Invasive Gynecol.

[7] Fascilla FD, Resta L, Cannone R (2020). Resectoscopic metroplasty with uterine septum excision: a histologic analysis of the uterine septum.. J Minim Invasive Gynecol.

[8] Grimbizis GF, Gordts S, Di Spiezio Sardo A (2013). The ESHRE/ESGE consensus on the classification of female genital tract congenital anomalies.. Hum Reprod.

[9] Grynberg M, Gervaise A, Faivre E (2012). Treatment of twenty-two patients with complete uterine and vaginal septum.. J Minim Invasive Gynecol.

[10] Ignatov A, Costa SD, Kleinstein J (2008). Reproductive Outcome of Women with Rare Müllerian Anomaly: Report of 2 Cases.. J Minim Invasive Gynecol.

[11] Lin K, Zhu X, Xu H (2009). Reproductive outcome following resectoscope metroplasty in women having a complete uterine septum with double cervix and vagina.. Int J Gynecol Obstet.

[12] Louden ED, Awonuga AO, Gago LA (2015). Rare Müllerian Anomaly: Complete Septate Uterus with Simultaneous Longitudinal and Transverse Vaginal Septa.. J Pediatr Adolesc Gynecol.

[13] Ludwin A, Ludwin I, Pityński K (2013). Differentiating between a double cervix or cervical duplication and a complete septate uterus with longitudinal vaginal septum.. Taiwan J Obstet Gynecol.

[14] Noventa M, Spagnol G, Marchetti M (2022). Uterine Septum with or without Hysteroscopic Metroplasty: Impact on Fertility and Obstetrical Outcomes-A Systematic Review and Meta-Analysis of Observational Research.. J Clin Med.

[15] Parodi L, Hoxhaj I, Dinoi G (2022). Complete uterine septum, double cervix and vaginal septum (U2b C2 V1): hysteroscopic management and fertility outcomes-A systematic literature review.. J Clin Med.

[16] Parsanezhad ME, Alborzi S, Zarei A (2006). Hysteroscopic metroplasty of the complete uterine septum, duplicate cervix, and vaginal septum.. Fertil Steril.

[17] Rikken JFW, Kowalik CR, Emanuel MH (2021). Septum resection versus expectant management in women with a septate uterus: an international multicentre open-label randomized controlled trial.. Hum Reprod.

[18] Seet MJ, Lau MSK, Chan JKY (2015). Management of complete vagino-uterine septum in patients seeking fertility: Report of two cases and review of literature.. Gynecol Minim Invasive Ther.

[19] Sejelfot EB, Nytun OE, Kjotrod SB (2020). A septate uterus with double cervix during two pregnancies: pregnancy outcome before and after cervix sparing metroplasty. A case report. Fact Views Vis ObGyn.

[20] Tomaževič T, Ban-Frangež H, Virant-Klun I (2010). Septate, subseptate and arcuate uterus decrease pregnancy and live birth rates in IVF/ICSI.. Reprod Biomed Online.

[21] Wang JH, XU KH, Lin J (2009). Hysteroscopic septum resection of complete septate uterus with cervical duplication, sparing the double cervix in patients with recurrent spontaneous abortions or infertility.. Fertil Steril.

